# Subacute toxicity and stability of Soshiho-tang, a traditional herbal formula, in Sprague–Dawley rats

**DOI:** 10.1186/1472-6882-12-266

**Published:** 2012-12-27

**Authors:** In Sik Shin, Mee Young Lee, Yongbum Kim, Chang Seob Seo, Jung Hun Kim, Hyeun Kyoo Shin

**Affiliations:** 1 Basic Herbal Medicine Research Group, Korea Institute of Oriental Medicine, 483 Expo-ro, Yusung-gu, Daejeon, 305-811, Republic of Korea; 2 Department of Nonclinical Studies, Korea Institute of Toxicology, 141 Gajeong-ro, Yusung-gu, Daejeon, 305-343, Republic of Korea

**Keywords:** Herbal formula, Soshiho-tang, Toxicity, Stability, Rat

## Abstract

**Backgroud:**

Soshiho-tang (SST, Xiao-chai-hu-tang in Chinese and Sho-saiko-to in Japanese), an oriental herbal formula, is used for treatment of chronic liver diseases. Although many researchers have studied the pharmacological properties of SST, information about its safety and toxicity is limited. Therefore, we evaluated the potential safety of SST in Sprague–Dawley rats over a period of 4-weeks.

**Methods:**

The SST was administered once daily by gavage to male and female rats at doses of 0, 500, 1000 and 2000 mg/kg/day for 4 weeks. We measured the body weight, mortality, food consumption, ophthalmoscopy, urinalysis, hematology, serum biochemistry, gross pathological findings, absolute/relative organ weights and histopathology. In addition, we analyzed the component of SST and measured the stability of its component in SST according to study periods using high performance liquid chromatography.

**Results:**

The SST treatment did not result in any toxicologically significant changes in mortality, food consumption, ophthalmoscopy, urinalysis, hematology, serum biochemistry, gross pathological findings, absolute/relative organ weights and histopathology, except for salivation and reduction in body weight in the 2000 mg/kg/day male group. These findings in the 2000 mg/kg/day male group are considered toxicologically insignificant because they are not accompanied by other pathological findings, including in hematology, serum biochemistry and histopatholgy, and they do not exhibit a dose–response relationship. SST is detected three components including liquiritin, baicalin, and glycyrrhizin. In addition, there were not observed the significant differences among the contents of three components in SST according to storage periods.

**Conclusion:**

These results indicate that SST may be a safe material. Based on these results, the no-observed-adverse-effect level was more than 2000 mg/kg for both genders.

## Background

Herbal formulas are traditionally used in Korea, China and Japan. The preparation of formulas is a form of oriental herbology, in which herbs are combined to achieve greater efficacy than is possible with individual herbs alone. Such herbal formulas are used for treatment of various disorders that can be considered chronic by clinical standards. Many researchers have investigated the properties of herbal formulas in a range of experimental systems. In addition, patients and physicians use herbal formulas to supplement the treatment of diseases with Western-style medicines [1,2]. However, the toxicity and quality control of herbal formulas for public consumption are one of increasing concern because of the lack of scientific evidence on their safety. Indeed, few studies have explored the safety and toxicity of herbal formulas, generating concerns regarding their potential adverse effects [3,4]. This has led to an urgent need to evaluate the safety of herbal formulas through basic science or clinical trials, to provide the essential information required for their clinical use.

Soshiho-tang (SST; Xiao-chai-hu-tang in Chinese and Sho-saiko-to in Japanese), an oriental herbal formula, is commonly used in Korea, China and Japan. It consist of seven herbs: *Bupleurum falcatum, Pinellia ternate, Scutellaria baicalensis, Zizyphus jujuba, Panax ginseng, Glycyrrhiza uralensis and Zingiber officinale*. According to previous studies, SST exhibits various pharmacological properties including anti-inflammatory [5], antioxidant [6], immunomodulatory [7], hepatoprotective [8], anti-hepatic fibrotic [9], and antitumoral effects [10]. In particular, SST has been shown in both basic and clinical studies to have powerful protective effects via modulation of the immune response to hepatic viral infection in diseases such as chronic viral hepatitis C [11-13]. Indeed, SST is traditionally used for treatment of chronic hepatitis, liver cirrhosis and various chronic inflammatory diseases [14].

Although SST has been found to have a variety of pharmacological effects, few scientific studies have examined its safety and toxicity. Therefore, we evaluated the toxicity of a 4-week repeated oral dose of SST in Sprague–Dawley (SD) rats to establish its safety and toxicity profiles of SST. In addition, we analyzed the components in SST by high performance liquid chromatogram (HPLC) and evaluated the stability of SST according to storage periods by measuring the contents of components. The present study was conducted according to guidelines established by the Organization for Economic Cooperation and Development (OECD) for the testing of chemicals in accordance with the current regulations for Good Laboratory Practice Regulations.

## Methods

### Preparation of SST

The SST consisting of seven herbal medicines (Table 1) and each crude drug was purchased from Omniherb (Yeongcheon, Korea) and HMAX (Jecheon, Korea). The origin of materials was confirmed taxonomically by Prof. Je-Hyun Lee of Dongguk University (Gyeongju, Republic of Korea) and Prof. Young-Bae Seo of Daejeon University (Daejeon, Republic of Korea). A decoction of SST was prepared in our laboratory (Table 1, 38.46 kg) from a mixture of chopped crude herbs, extracted in distilled water at 100°C for 2 h in an herb extractor (COSMOS-660, Kyungseo Machine Co., Incheon, Korea). The solution was filtration using a standard sieve (No. 270, 53 μm) and freeze-dried (6.30 kg). The yield of SST extract was 16.37%. The yield of Soshiho-tang extract was 16.37%. For HPLC analysis, lyophilized SST extract (200 mg) was dissolved in distilled water (20 mL). The solution was filtered using a syringe filter (0.2 μm, Woongki Science, Seoul, Korea).

**Table 1:** The composition of SST

**Latin name**	**Amount (g)**	**Ratio**	**Company of purchase**	**Source**
Bupleuri Radix	11.25	6	HMAX	China
Pinelliae Tuber	7.5	4	HMAX	Jeongseon, Korea
Scutellariae Radix	3.75	2	HMAX	China
Ginseng Radix Alba	3.75	2	Omniherb	Geumsan, Korea
Zizyphi Fructus	3.75	2	Omniherb	Yeongcheon, Korea
Zingiberis Rhizoma Crudus	3.75	2	Omniherb	Yeongcheon, Korea
Glycyrrhizae Radix	1.875	1	HMAX	China
Total	35.625			

### Reagents

The two flavonoids, liquiritin and baicalin, and one triterpenoid saponin glycoside, glycyrrhizin were purchased from Wako (Osaka, Japan). The purity of each component was determined to be above 98% by HPLC analysis. HPLC-grade reagents, methanol, acetonitrile, and water were obtained from J.T.Baker (Phillipsburg, NJ, USA). Glacial acetic acid was of analytical reagent grade, procured from Junsei (Tokyo, Japan).

### Linearity, limits of detection (LOD) and quantification (LOQ)

Standard stock solutions of liquiritin, baicalin, and glycyrrhizin were dissolved in methanol in the concentration of 1.0 mg/mL and stored below 4°C. Working standard solutions were prepared by serial dilution of stock solutions with methanol. Calibration curves of three compounds were calculated by peak areas of standard solutions in the concentration of 3.91–250.00 μg/mL. The LOD and LOQ data obtained under the chromatographic conditions used in the present study were determined using signal-to-noise (S/N) ratios of 3 and 10, respectively.

### Stability

The stabilities of liquiritin, baicalin, and glycyrrhizin were determined by six injections with sample solution during 10 days (0, 1, 2, 5, 7, and 10 day, respectively). In addition, we measure the stabilities of liquiritin, baicalin, and glycyrrhizin in SST once a weeks for 4 weeks.

### Instruments and chromatographic conditions

A Shimadzu LC-20A HPLC system (Shimadzu Co., Kyoto, Japan) consisting of a solvent delivery unit, an on-line degasser, a column oven, an autosampler, and a PDA detector. The data processor employed LCsolution software (Version 1.24). The analytical column used was a Gemini C18 (250×4.6 mm; particle size 5 μm, Phenomenex, Torrance, CA, USA). The mobile phases consisted of (A) 1.0%, v/v, aqueous acetic acid and (B) acetonitrile with 1.0%, v/v, acetic acid. The gradient flow was as follows: (A)/(B)=85/15 (0 min) → (A)/(B)=60/40 (15 min) → (A)/(B)=45/55 (40 min) → (A)/(B)= 0/100 (50 min; hold for 5 min) → (A)/(B)= 85/15 (55 min; hold for 15 min). Column temperature was maintained at 30°C. The analysis was carried out at a flow rate of 1.0 mL/min with PDA detection at 254 and 275 nm. The injection volume was 10 μL.

### Animal

Twenty 4-weeks-old SD rats of each sex were obtained from Orient Bio Co. (Seoul, Korea), a specific pathogen-free facility, and used after 2 weeks of quarantine and acclimatization. The animals were housed in a room maintained at 22 ± 3°C under a relative humidity of 50 ± 20% with artificial lighting from 08:00 to 20:00 and 12–15 air changes per hour. Three animals were kept in stainless-steel wire-mesh cages and allowed sterilized tap water and commercial rodent chow (PMI Nutrition International, Richmond, USA) *ad libitum*. This study was approved by Korea Institute of Oriental Medicine Institutional Animal Care and Use Committee and was performed at the Korea Institute of Toxicology (Daejeon, Korea) and conducted according to the guidance of the Institutional Animal Care and Use Committee in Korea Institute of Toxicology (KRICT) (accredited by AAALAC International, 1998) under the Good Laboratory Practice Regulations for Nonclinical Laboratory Studies.

### Experimental groups and treatment

Healthy male and female rats were assigned to four groups using Path/Tox System 4.2.2 (Xybion Medical Systems Corporation, USA). Each group consisted of five rats of each sex. Before performing this study, we conducted the acute toxicity study according to OECD guideline. As results of acute toxicity study, SST did not exhibit any adverse effects at dose level of up to 2000 mg/kg in both genders. Based on these results, we established 2000 mg/kg/day as the maximum dose, with 500 mg/kg/day and 1000 mg/kg/day as low and medium doses, respectively. The SST was prepared fresh on each treatment day the vehicle-only control group received an equal volume of distilled water. Because oral administration is the clinically intended route for SST, it was administered by oral gavage in the present study. The daily dose (10 ml/kg body weight) of SST was calculated based on the most recently recorded body weights of individual animals.

### General observations

Clinical signs and mortality were recorded twice a day (before and after treatment) throughout the study period. All clinical signs were recorded individually for type, observation day/time and duration using Path/Tox System 4.2.2 (Xybion Medical Systems Corporation, USA). The body weight of each rat was measured at the initiation of treatment and once a week during the study period. Food consumption was measured at the start of treatment and weekly throughout. Daily food consumption was determined by measuring the weight of chow supplied and remaining each day. External eye examination was carried out during the last week of treatment with an indirect binocular ophthalmoscope (IO-H, Neitz Instrument Co., Japan), and the appearance of the conjunctiva, sclera, cornea, lens, and iris of each eye was recorded.

### Urinalysis, hematology and serum biochemistry

During the last week of treatment, urinalysis was conducted on samples collected overnight using a Multistix 10 SG (Bayer, USA) and urine chemical analyzer (Clinitek-500, USA). Analysis included volume, specific gravity (SG), pH, protein, ketone body (KET), occult blood (BLO), glucose, bilirubin (BIL), nitrite (NIT), urobilinogen (URO) and sediment.

Animals were fasted overnight prior to blood collection or necropsy. Blood was drawn from the posterior vena cava under isoflurane anesthesia. Samples were collected in CBC bottles containing EDTA-2K (Sewon Medical Co., Republic of Korea), and were analyzed to determine red blood cell count (RBC), white blood cell count (WBC), differential WBC count, hemoglobin concentration (HGC), hematocrit (HCT), mean corpuscular volume (MCV), mean corpuscular hemoglobin (MCH), mean corpuscular hemoglobin concentration (MCHC), platelet (PLT) and reticulocyte (RET) using an ADVIA120 Hematology System (Bayer, USA). Prothrombin time (PT) and activated partial thromboplastin time (APTT) were determined in blood samples treated with 3.2% sodium citrate using a coagulometer (ACL 300 plus, Instrumentation Laboratory, Italy).

For serum biochemistry, blood samples were centrifuged at 3000 rpm for 10 min and analyzed with an autoanalyzer (Toshiba 200FR NEO, Toshiba Co., Japan). The analysis included alanine aminotransferase (ALT), aspartate aminotransferase (AST), alkaline phosphatase (ALP), gamma glutamyl transpeptidase (GGT), blood urea nitrogen (BUN), creatinine (CREA), creatine kinase (CK), glucose (GLU), total cholesterol (TCHO), albumin (ALB), albumin/globulin ratio (A/G), total protein (TP), triglyceride (TG), total bilirubin (TBIL), phospholipids (PL), sodium (Na), potassium (K), calcium (Ca), chloride (Cl) and inorganic phosphorus (IP).

### Necropsy

All surviving animals were anesthetized with isofluorane and sacrificed by aortic exsanguination prior to necropsy. Complete gross postmortem examinations were performed on all animals. Absolute organ weights were measured and relative organ weights (organ-to-body weight ratios) were calculated for the following organs: brain, pituitary gland, adrenal gland, liver, spleen, kidneys, heart, thymus, lung, salivary gland, thyroids, testes, ovaries, epididymides, seminal vesicle, prostate and uterus.

### Histopathological examination

Liver and kidney samples were fixed in 10% neutral buffered formalin, embedded in paraffin, sectioned at 4 μm and stained with hematoxylin (Sigma MHS-16) and eosin (Sigma HT110-1-32). Tissues were subsequently mounted and coverslipped using Dako mounting medium (Invitrogen, USA).

### Statistical analysis

Data collected during the study were examined for the variance homogeneity using Bartlett’s test. When Bartlett’s test indicated no significant deviation from the variance homogeneity, a one-way ANOVA was performed at α=0.05. When significance was noted, a multiple comparison test (Dunnett’s test) was conducted to determine which pairs of groups were significantly different. Where significant deviations from variance homogeneity were observed, a nonparametric comparison test (Kruskal–Wallis test) was conducted. When a significant difference was observed in the Kruskal–Wallis test, the Dunn’s Rank Sum test was conducted to determine the specific pairs. Statistical analyses were performed using the Path/Tox System (ver. 4.2.2). The level of significance was taken as *p*<0.05 or 0.01.

## Results

### Clinical signs and mortality

In female rats, no clinical signs were observed (data not shown). By contrast, in male rats, salivation was observed in all animals in the 2000 mg/kg group, and in one case in the 500 mg/kg and 1000 mg/kg groups. Death did not occur in either sex at any dose, or in the vehicle-only control group.

### Body weight changes and food consumption

**Figure 1:**
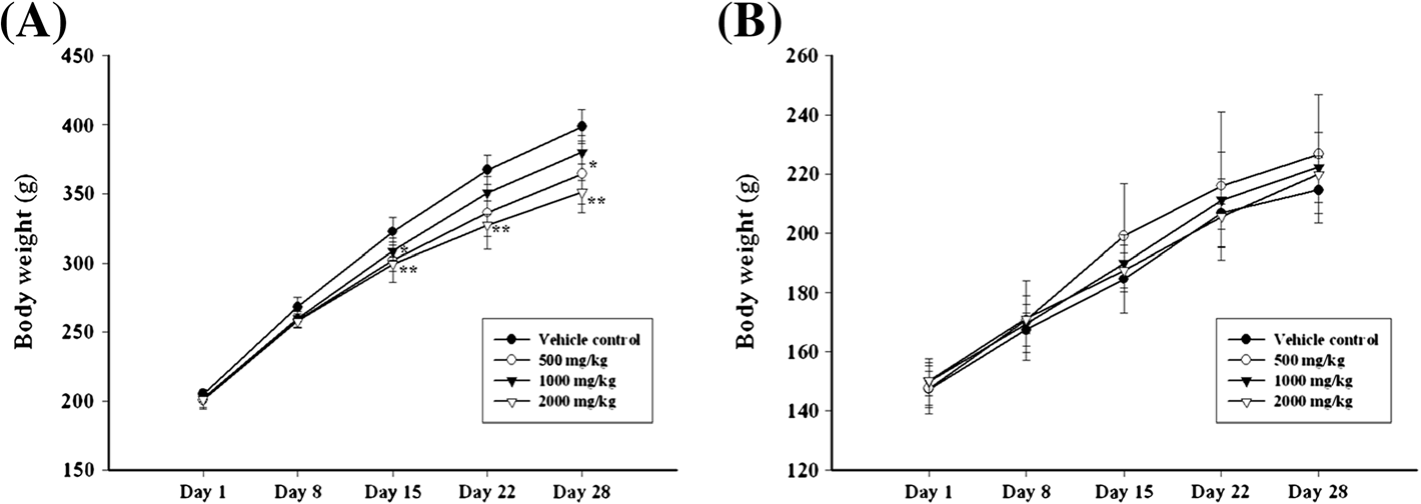
**Body weight changes of male and female rat treated with SST for 4-weeks.** (**A**) body weight changes of males, (**B**) body weight changes of female. ^*^,^**^ Indicate a significant differences at P < 0.05 and P < 0.01 levels, respectively, when compared with the vehicle control group.

There was no significant difference in body weights between the vehicle-only control group and the treatment groups in females (Figure 1). However, for the 2000 mg/kg male group, body weight was significantly reduced on days 15, 22 and 28, and for the 1000 mg/kg male group, there was a significant reduction on day 28. No significant differences were observed in food consumption between the vehicle-only control and treatment groups for either sex (data not shown). In addition, ophthalmologic examinations did not reveal treatment related ocular lesions in any of the animals (data not shown).

### Necropsy findings

There were no treatment-related gross pathological changes at necropsy, with the exception of a size reduction in epididymides (n = 1), prostate (n = 1) and testes (*n* = 1) in the 1000 mg/kg male group (data not shown). In females, no gross pathological findings were observed in any group.

For absolute organ weights, the 2000 mg/kg male group showed significant reductions in pituitary gland, liver, lung and thyroid/parathyroid (data not shown). In female groups, there was no significant difference in absolute organ weights between the vehicle-only control and treatment groups (data not shown). Finally, there was no significant difference in relative organ weights between the vehicle-only control and treatment groups for either sex (Table 2).

**Table 2:** Relative organ weights male rats treated with SST for 4 weeks

**Dose (mg/kg/day)**	**0**	**500**	**1000**	**2000**
Males				
Brain	0.54 ± 0.04 ^a^	0.58 ± 0.05	0.54 ± 0.03	0.59 ± 0.01
Pituitary gland	0.003 ± 0.0003	0.003 ± 0.0006	0.003 ± 0.0003	0.002 ± 0.0005
Liver	3.25 ± 0.12	3.15 ± 0.21	3.06 ± 0.15	3.05 ± 0.15
Spleen	0.19 ± 0.01	0.19 ± 0.03	0.20 ± 0.03	0.18 ± 0.03
Heart	0.33 ± 0.02	0.34 ± 0.02	0.34 ± 0.02	0.34 ± 0.02
Thymus	0.14 ± 0.03	0.16 ± 0.05	0.15 ± 0.03	0.11 ± 0.01
Salivary glands	0.17 ± 0.01	0.17 ± 0.02	0.17 ± 0.01	0.19 ± 0.02
Seminal vesicle	0.36 ± 0.08	0.41 ± 0.11	0.33 ± 0.06	0.38 ± 0.04
Prostate	0.13 ± 0.02	0.13 ± 0.01	0.13 ± 0.02	0.12 ± 0.02
Kidneys	0.85 ± 0.06	0.82 ± 0.03	0.83 ± 0.05	0.83 ± 0.05
Adrenal glands	0.017 ± 0.002	0.015 ± 0.002	0.016 ± 0.002	0.017 ± 0.003
Testes	0.89 ± 0.04	0.88 ± 0.13	0.88 ± 0.14	0.87 ± 0.05
Epididymides	0.27 ± 0.03	0.27 ± 0.05	0.27 ± 0.02	0.26 ± 0.02
lung	0.41 ± 0.03	0.40 ± 0.04	0.39 ± 0.02	0.38 ± 0.03
Thyroid/Parathyroid	0.006 ± 0.001	0.005 ± 0.001	0.006 ± 0.001	0.005 ± 0.001
Females				
Brain	0.90 ± 0.05	0.85 ± 0.10	0.87 ± 0.03	0.87 ± 0.02
Pituitary gland	0.006 ± 0.0009	0.005 ± 0.0005	0.006 ± 0.0010	0.005 ± 0.0009
Liver	3.25 ± 0.16	3.23 ± 0.13	3.25 ± 0.26	3.36 ± 0.10
Spleen	0.22 ± 0.04	0.21 ± 0.02	0.23 ± 0.03	0.24 ± 0.02
Heart	0.36 ± 0.02	0.35 ± 0.01	0.37 ± 0.02	0.37 ± 0.02
Thymus	0.19 ± 0.02	0.18 ± 0.03	0.21 ± 0.04	0.21 ± 0.04
Salivary glands	0.19 ± 0.02	0.19 ± 0.01	0.19 ± 0.01	0.21 ± 0.02
Kidneys	0.85 ± 0.04	0.85 ± 0.02	0.85 ± 0.08	0.94 ± 0.07
Adrenal glands	0.034 ± 0.004	0.029 ± 0.001	0.032 ± 0.005	0.034 ± 0.004
Ovaries	0.042 ± 0.004	0.040 ± 0.005	0.041 ± 0.009	0.040 ± 0.005
Lung	0.53 ± 0.02	0.51 ± 0.03	0.51 ± 0.05	0.50 ± 0.04
Thyroid/parathyroid	0.007 ± 0.002	0.006 ± 0.001	0.007 ± 0.001	0.008 ± 0.001
Uterus/cervix	0.23 ± 0.03	0.26 ± 0.12	0.23 ± 0.07	0.32 ± 0.11

^a^ Values are presented as mean ± SD.

### Hematology serum biochemistry and urinalysis

No significant differences were noted with hematology tests in any female group. In contrast, the 2000 mg/kg male group showed a significant increase in percent neutrophils (Table 3). However, there was no significant difference in serum biochemical (Table 4) and urinalysis values (data not shown) between the vehicle-only control and treatment groups.

**Table 3:** Hematological values of animals treated with SST for 4 weeks

**Dose (mg/kg/day)**	**0**	**500**	**1000**	**2000**
Male rat				
WBC (10^3^/μL)	11.05 ± 2.35^a^	12.76 ± 1.78	12.68 ± 3.36	9.14 ± 2.40
Reticulocyte (%)	2.7 ± 0.42	2.7 ± 0.20	3.0 ± 0.73	2.3 ± 0.23
Neutrophils (%)	9.18 ± 2.34	11.28 ± 3.40	10.32 ± 2.35	14.60 ± 2.78*
Lymphocytes (%)	86.5 ± 3.26	81.9 ± 3.25	84.6 ± 2.67	80.1 ± 3.62*
Eosinophils (%)	0.8 ± 0.40	0.6 ± 0.09	0.6 ± 0.22	0.5 ± 0.12
Monocytes (%)	2.0 ± 0.59	2.7 ± 0.66	2.6 ± 0.63	2.6 ± 0.56
Basophils (%)	0.8 ± 0.20	1.1 ± 0.22	1.0 ± 0.21	1.3 ± 0.35
Large unstained cells (%)	0.7 ± 0.24	2.4 ± 2.97	0.9 ± 0.16	1.0 ± 0.34
RBC (10^6^/μL)	8.04 ± 0.16	8.33 ± 0.43	8.15 ± 0.38	8.22 ± 0.55
Hemoglobin (g/dl)	15.8 ± 0.51	16.2 ± 0.58	16.1 ± 0.52	15.8 ± 0.49
Hematocrit (%)	47.7 ± 2.12	48.3 ± 1.98	48.1 ± 1.75	48.0 ± 1.76
MCV (fL)	59.2 ± 1.64	58.1 ± 1.68	59.0 ± 1.34	58.5 ± 1.79
MCH (pg)	19.6 ± 0.32	19.4 ± 0.61	19.8 ± 0.38	19.3 ± 0.75
MCHC (g/dl)	33.1 ± 0.37	33.5 ± 0.73	33.5 ± 0.44	32.9 ± 0.31
Platelet (10^3^/μL)	1305 ± 225	1164 ± 40	1198 ± 67	1178 ± 66
PT (sec)	17.5 ± 0.86	17.2 ± 0.45	16.5 ± 0.87	16.7 ± 0.62
APTT (sec)	16.3 ± 0.80	16.7 ± 0.80	15.4 ± 1.11	16.3 ± 0.49
Female rat				
WBC (10^3^/μL)	8.07 ± 1.25	8.17 ± 2.38	8.27 ± 1.09	6.75 ± 0.56
Reticulocyte (%)	2.2 ± 0.38	2.7 ± 0.39	2.4 ± 0.58	2.5 ± 0.55
Neutrophils (%)	13.12 ± 6.54	15.92 ± 7.16	15.58 ± 5.63	13.20 ± 3.05
Lymphocytes (%)	81.6 ± 6.97	78.6 ± 7.19	79.2 ± 5.09	82.7 ± 2.81
Eosinophils (%)	1.0 ± 0.15	1.3 ± 0.38	1.1 ± 0.15	0.8 ± 0.17
Monocytes (%)	2.2 ± 0.86	2.6 ± 0.40	2.4 ± 0.61	1.7 ± 0.35
Basophils (%)	0.9 ± 0.21	0.8 ± 0.25	0.7 ± 0.13	0.9 ± 0.13
Large unstained cells (%)	1.2 ± 0.42	0.9 ± 0.23	1.0 ± 0.08	0.8 ± 0.26
RBC (10^6^/μL)	8.24 ± 0.33	8.28 ± 0.16	8.18 ± 0.20	8.14 ± 0.13
Hemoglobin (g/dl)	15.9 ± 0.55	15.7 ± 0.44	16.0 ± 0.13	15.7 ± 0.19
Hematocrit (%)	46.4 ± 1.62	46.4 ± 1.06	47.0 ± 0.43	46.2 ± 0.73
MCV (fL)	56.4 ± 0.88	56.0 ± 1.67	57.5 ± 1.25	56.8 ± 0.49
MCH (pg)	19.3 ± 0.43	19.0 ± 0.54	19.6 ± 0.50	19.4 ± 0.35
MCHC (g/dl)	34.3 ± 0.28	33.9 ± 0.38	34.1 ± 0.40	34.1 ± 0.49
Platelet (10^3^/μL)	1242 ± 76	1595 ± 430	1161 ± 100	1132 ± 32
PT (sec)	17.6 ± 0.43	18.1 ± 0.26	17.6 ± 0.59	18.3 ± 0.73
APTT (sec)	13.9 ± 0.39	15.4 ± 0.86	14.3 ± 1.10	14.8 ± 0.75

APTT, activated partial thromboplastin time; MCV, mean corpuscular volume; MCH, mean corpuscular hemoglobin; MCHC, mean corpuscular hemoglobin concentration; PT, prothrombin time.

^a^ Values are presented as mean ± SD.

* Indicate a significant difference at P < 0.05 level, when compared with the vehicle control group.

**Table 4:** Serum biochemical values of animals treated with SST for 4 weeks

**Dose (mg/kg/day)**	**0**	**500**	**1000**	**2000**
**Male rat**				
Glucose (mg/dL)	120.0 ± 14.95	131.6 ± 18.66	133.8 ± 24.86	122.4 ± 6.90
BUN (mg/dL)	14.0 ± 1.58	13.5 ± 1.09	12.9 ± 1.90	12.9 ± 1.60
Creatinine (mg/dL)	0.57 ± 0.05	0.55 ± 0.05	0.55 ± 0.03	0.58 ± 0.04
Total protein (g/dL)	6.42 ± 0.13	6.54 ± 0.17	6.19 ± 0.29	6.48 ± 0.20
Albumin (g/dL)	4.08 ± 0.06	4.12 ± 0.12	3.99 ± 0.09	4.09 ± 0.09
Albumin/globulin ratio	1.75 ± 0.11	1.70 ± 0.08	1.83 ± 0.15	1.71 ± 0.05
Total cholesterol (mg/dL)	52.0 ± 7.75	57.0 ± 8.51	57.6 ± 18.77	50.8 ± 6.42
Triglycerides (mg/dL)	32.1 ± 9.98	31.4 ± 9.83	36.5 ± 8.41	36.0 ± 9.96
Phospholipid (mg/dL)	86 ± 10.0	90 ± 5.1	87 ± 19.6	87 ± 11.3
AST (IU/L)	136.1 ± 20.74	152.0 ± 33.87	119.8 ± 14.15	157.6 ± 32.13
ALT (IU/L)	28.4 ± 2.67	36.2 ± 11.11	30.5 ± 3.22	32.7 ± 2.25
Total bilirubin (mg/dL)	0.13 ± 0.010	0.13 ± 0.004	0.12 ± 0.015	0.113 ± 0.015
ALP (IU/L)	590.0 ± 84.05	506.1 ± 104.95	521.4 ± 94.21	557 ± 52.56
Creatine kinase (IU/L)	872 ± 275	935 ± 321	759 ± 156	989 ± 327
Female				
Glucose (mg/dL)	100.3 ± 28.10	89.1 ± 18.43	98.9 ± 10.13	108.1 ± 18.59
BUN (mg/dL)	18.7 ± 1.87	17.7 ± 1.89	15.6 ± 2.22	15.3 ± 2.26
Creatinine (mg/dL)	0.62 ± 0.05	0.65 ± 0.05	0.58 ± 0.07	0.60 ± 0.03
Total protein (g/dL)	6.96 ± 0.11	6.94 ± 0.16	6.79 ± 0.14	6.98 ± 0.21
Albumin (g/dL)	4.47 ± 0.15	4.45 ± 0.09	4.34 ± 0.04	4.48 ± 0.10
Albumin/globulin ratio	1.79 ± 0.11	1.79 ± 0.08	1.78 ± 0.07	1.80 ± 0.13
Total cholesterol (mg/dL)	73.2 ± 14.96	65.6 ± 8.23	60.2 ± 7.36	56.0 ± 17.18
Triglycerides (mg/dL)	27.3 ± 3.67	25.6 ± 1.54	29.1 ± 7.61	25.2 ± 5.98
Phospholipid (mg/dL)	124 ± 19.4	111 ± 9.8	106 ± 11.6	104 ± 21.5
ASP (IU/L)	160.3 ± 25.73	142.6 ± 9.32	155.9 ± 27.99	140.9 ± 33.54
ALT (IU/L)	25.7 ± 2.47	30.0 ± 5.14	25.3 ± 3.46	26.7 ± 4.66
Total bilirubin (mg/dL)	0.13 ± 0.014	0.13 ± 0.014	0.12 ± 0.011	0.13 ± 0.010
ALP (IU/L)	413.6 ± 61.29	432.5 ± 86.29	379.4 ± 40.70	383.6 ± 103.75
Creatine kinase (IU/L)	1127 ± 288	966 ± 196	1044 ± 263	947 ± 411

ALP, alkaline phosphatase; AST, aspartate aminotransferase; ALT, alkaline phosphatase; BUN, blood urea nitrogen.

^a^ Values are presented as mean ± SD.

### Histopathological examination

Administration of SST did not induce histopathological changes in liver or kidney at any dose level (data not shown).

### Linearity, range, LOD and LOQ

The linearity of the peak area (y) versus concentration (x, μg/mL) curve for reference compounds such as liquiritin, baicalin, and glycyrrhizin was used to calculate the contents of the main components in Soshiho-tang. The correlation coefficients (*R*^2^) of the calibration curves for seven constituents were ≥0.9993. The regression equations were Y = 20031.34x – 19996.27 for liquiritin, Y = 38806.45x – 79723.75 for baicalin, and Y = 8959.42x – 15143.09 for glycyrrhizin. These results showed that the calibration curve was a good linearity. The LODs and LOQs were 0.09–0.21 μg/mL and 0.29–1.37 μg/mL, respectively.

### Stability of SST

The stability test of liquiritin, baicalin, and glycyrrhizin was evaluated using the sample solution for 10 days and 4 weeks. In Table 5, sample solution retained a content of 93.66–101.37% as compared with the initial content at 0 day. The RSD values of contents about three compounds in sample solution were within 1.59%. In addition, there was no observed the significant difference in contents of three components according storage periods of 4 weeks (Table 6).

**Table 5:** Stability of three components for 10 days in the SST (n=3)

**Compound**	**Day**											
	**0**		**1**		**2**		**5**		**7**		**10**	
	**Mean ± SD (mg/g)**	**RSD (%)**	**Mean ± SD (mg/g)**	**RSD (%)**	**Mean ± SD (mg/g)**	**RSD (%)**	**Mean ± SD (mg/g)**	**RSD (%)**	**Mean ± SD (mg/g)**	**RSD (%)**	**Mean ± SD (mg/g)**	**RSD (%)**
Liquiritin	2.8±0.04	1.59	2.8±0.00	0.03	2.8±0.01	0.34	2.8±0.01	0.44	2.6±0.00	0.05	2.7±0.01	0.50
Baicalin	60.9±0.04	0.06	60.9±0.04	0.07	60.0±0.26	0.43	61.0±0.17	0.28	60.8±0.07	0.11	60.6±0.02	0.04
Glycyrrhizin	2.3±0.01	0.63	2.3±0.03	1.14	2.3±0.02	0.65	2.3±0.00	0.07	2.4±0.00	0.17	2.3±0.01	0.45

**Table 6:** Contents of three components in the SST for 4 weeks by HPLC (n=3)

**Compound**	**0 week**				**1 week**			**4 week**		
	**Mean (mg/g)**	**SD**	**RSD (%)**	**Mean (mg/g)**	**SD**	**RSD (%)**	**Mean (mg/g)**	**SD**	**RSD (%)**	
Liquiritin	2.71	0.01	0.42	2.66	0.03	0.98	2.57	0.02	0.82
Baiclin	61.58	0.02	0.04	60.16	0.28	0.46	57.19	0.38	0.66
Glycyrrhizin	2.33	0.00	0.11	2.54	0.01	0.59	2.43	0.03	1.43

### HPLC analysis of SST

**Figure 2:**
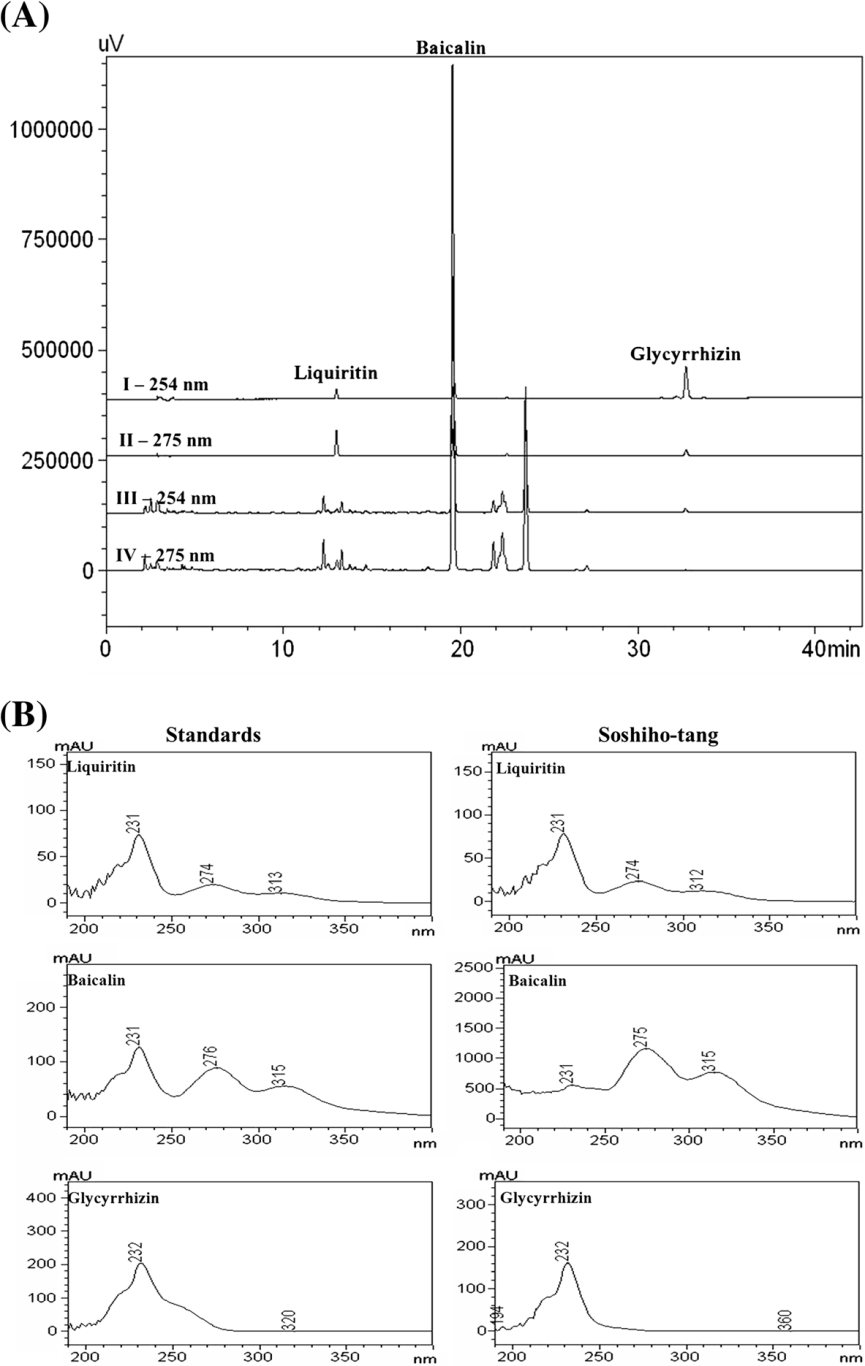
HPLC chromatogram of the standard mixture and SST with PDA detector (A) and UV–vis absorption spectra of compounds (B).

Our analysis method was applied to the simultaneous determination of three compounds, liquiritin, baicalin, and glycyrrhizin in Soshiho-tang. Figure 2 show chromatograms of reference components and water extract of Soshiho-tang and UV spectrum of these compounds. The retention times of the three compounds were 13.00, 19.88, and 33.42 min for liquiritin, baicalin, and glycyrrhizin, respectively. The contents of three components identified at 0, 1, and 4 weeks in Soshiho-tang were not observed significant differences. These results were summarized in Table 6.

## Discussion

In current biomedical research, many studies of therapeutic materials have been based on evidence-based medicine (EBM). EBM aims to apply knowledge gained from rigorous scientific research to find solutions to problematic clinical issues [15]. In EBM, evidence of therapeutic efficacy is evaluated by such methods as toxicology, pharmacokinetics, clinical trials and systematic reviews [16]. Herbal medicines have become increasingly popular in modern societies around the globe, and many researchers have conducted pharmacological studies on their constituents to strengthen the evidence of efficacy. However, herbal medicines are often not subjected to toxicity testing before human consumption because they are widely considered inherently safe. Recently, however, the potential for adverse effects of herbal medicines in clinical usage has become an important issue [17]. Therefore, toxicity studies are urgently needed to assess the safety of the wide array of herbal medicines in use. In this study, we evaluated the toxicity and safety of SST, which is used in Korea, China and Japan for treatment of liver diseases. This evaluation was done via a 4-week repeated-oral-dose toxicity study, carried out in accordance with OECD guidelines. In addition, we analyzed the components in SST and evaluated the changes in contents of its components according to storage period by HPLC. Present study showed that administration of SST did not have any adverse effects in SD rat of either sex, when given orally at 2000 mg/kg/day in both gender except for salivation and a reduction of body weight in 2000 mg/kg/day male group. The components in SST including liquiritin, biacalin and glycyrrhizin were no significant differences among its contents according storage periods.

There were no treatment-related effects with regard to food consumption, ophthalmoscopy, urinalysis, hematology, serum biochemistry, testosterone concentration, gross pathological findings, organ weight or histopathology, regardless of sex. However, all animals in the 2000 mg/kg/day male group exhibited excess salivation. This is considered to be related to SST treatment because it occurred in a high incidence and exhibited a dose response relationship. Furthermore, body weight was significantly decreased in the male group treated with 2000 mg/kg SST in a dose response fashion, suggesting that the reduction in body weight is caused by SST administration. However, the reduction in body weight of the 2000 mg/kg male group is considered to be of relatively little toxicological significance because the reduction caused is within the normal range, as established by our histological data and previous studies [18,19]. Further study is needed to determine whether these changes are induced by certain pharmacological properties or toxic effects of SST.

In absolute organ weights, significant differences were observed in the pituitary gland, liver, lung and thyroid/parathyroid of the 2000 mg/kg male group, compared with the vehicle-only control. However, this change was not considered an SST-induced abnormality because it was not observed in females of the same dose group and no dose dependent correlation was observed. Furthermore, this finding was not accompanied by relative weight changes and gross pathological findings.

To date, there were few reports on the potential side effects of SST. In Japan, some case reports have raised concerns regarding interstitial pneumonia and acute respiratory failure. Previous studies reported that patients took SST for treating chronic liver diseases, which caused interstitial pneumonia [20-22]. However, according to Lee et al. [14], the incidence of side effects is increased by other variables such as coadministration of interferon, the duration of SST treatment and increasing age of patients. Indeed, it was concluded that it is very difficult to determine whether the apparent side effects are causally linked to SST administration.

To investigate the chemical analysis and stability of SST according to periods, we have conducted HPLC analysis of SST. Liquiritin, baicalin and glycyrrhizin were detected in SST by HPLC analysis. These compounds have possessed various benefit effects such as antioxidative, antiinflammatory and antitumor effects proved by many experiments [23-25]. The contents of three components were not observed significant differences according to storage periods. These findings indicate that SST may be very safe material. In addition, this conclusion was supported by previous studies about toxicity studies of crude herbs including Bupleuri Radix, Scutellariae Radix and Ginseng Radix Alba [26-28]. Previous studies demonstrated that three crude herbs did not any toxic effects on repeated oral toxicity or genotoxicity studies.

## Conclusion

As mentioned previously, SST is recommended for treatment of chronic liver diseases, based on the results of various clinical trials and pharmacological studies. However, little information is available on the safety and toxicity of SST. The study demonstrates that SST does not induce specific adverse effects in SD rats when used in either sex at doses of up to 2000 mg/kg for 4 weeks. In human, the single dose of SST is about 35.625 g dried herbs, which is equivalent to 5.83 g of the SST extract (yield = 16.37%). Based on an average body weight of an adult of 60 kg, this dose for a 60 kg human is same as 97.2 mg of SST extract/kg [29,30]. This dosage is about 20-fold lower than high dose used in this study. Additionally, whether the increased salivation and reduced body weight were observed in males administered the highest dose (2000 mg/kg males) represent important side effects of SST can only be determined by further pharmacological and toxicity studies.

## Competing interest

The authors declare that they have no competing interest.

## Authors’ contributions

ISS, MYL and HKS participated in the design of the study data analyses and manuscript preparation. YBK conducted the histopathological examination of various tissues. CSS and JHK provided SST samples and analysis of three components in SST using HPLC. All authors read and approved the final manuscript.

## Pre-publication history

The pre-publication history for this paper can be accessed here:

http://www.biomedcentral.com/1472-6882/12/266/prepub

## References

[B1] Basch EM Servoss JC Tedrow UB Safety assurances for dietary supplements policy issues and new research paradigms J Herb Pharmacother 2005 5 3 15 16093231

[B2] Bent S Herbal medicine in the United States: review of efficacy, safety, and regulation: grand rounds at University of California, San Francisco Medical Center J Gen Intern Med 2008 23 854 859 10.1007/s11606-008-0632-y 18415652 PMC2517879

[B3] Ha H Lee JK Lee HY Seo CS Lee MY Huh JI Shin HK Genotoxicity assessment of a herbal formula, Ojeok-san J Ethnopharmacol 2011 135 586 589 10.1016/j.jep.2011.03.024 21419208

[B4] Shin IS Yu YB Seo CS Ha HK Lee MY Huang DS Kim JH Shin HK Subchronic toxicity of Sipjeondaebo-tang (SDT) in Sprague–Dawley Rats Regul Toxicol Pharmacol 2011 59 375 384 10.1016/j.yrtph.2010.09.018 20937344

[B5] Kusunose M Qiu B Cui T Hamada A Yoshioka S Ono M Miyamura M Kyotani S Nishioka Y Effect of Sho-saiko-to extract on hepatic inflammation and fibrosis in dimethylnitrosamine induced liver injury rats Biol Pharm Bull 2002 25 1417 1421 10.1248/bpb.25.1417 12419951

[B6] Shiota G Maeta Y Mukoyama T Yanagidani A Udagawa A Oyama K Yashima K Kishimoto Y Nakai Y Miura T Ito H Murawaki Y Kawasaki H Effects of Sho-Saiko-to on hepatocarcinogenesis and 8-hydroxy-2′-deoxyguanosine formation Hepatology 2002 35 1125 1133 10.1053/jhep.2002.33066 11981762

[B7] Chen MH Chen JC Tsai CC Wang WC Chang DC Tu DG Hsiesh HY The role of TGF-beta 1 and cytokines in the modulation of liver fibrosis by Sho-saiko-to in rat’s bile duct ligated model J Ethnopharmacol 2005 97 7 13 10.1016/j.jep.2004.09.040 15652268

[B8] Taira Z Yabe K Hamaguchi Y Hirayama K Kishimoto M Ishida S Ueda Y Effects of Sho-saiko-to extract and its components, Baicalin, baicalein, glycyrrhizin and glycyrrhetic acid, on pharmacokinetic behavior of salicylamide in carbon tetrachloride intoxicated rats Food Chem Toxicol 2004 42 803 807 10.1016/j.fct.2003.12.017 15046826

[B9] Chen MH Chen JC Tsai CC Wang WC Chang DC Lin CC Hsieh HY Sho-saiko-to prevents liver fibrosis induced by bile duct ligation in rats Am J Chin Med 2004 32 195 207 10.1142/S0192415X04001862 15315258

[B10] Zhu K Fukasawa I Furuno M Inhibitory effects of herbal drugs on the growth of human ovarian cancer cell lines through the induction of apoptosis Cynecol Oncol 2005 97 405 409 10.1016/j.ygyno.2004.12.063 15863137

[B11] Yamashiki M Nishimura A Nobori T Nakabayashi S Takagi T Inoue K Ito M In vitro effects of sho-saiko-to on production of granulocyte colony-stimulating factor by mononuclear cells from patients with chronic hepatitis C Int J Immunopharmacol 1997 19 381 385 10.1016/S0192-0561(97)00064-7 9568542

[B12] Ohtake N Yamamoto M Takeda S Aburada M Ishige A Watanabe K Inoue M The herbal Medicine Sho-saiko-to selectively inhibits CD8+ T-cell proliferation Eur J Pharmacol 2005 507 301 310 10.1016/j.ejphar.2004.11.037 15659321

[B13] Chang JS Wang KC Liu HW Chen MC Chiang LC Lin CC Sho-saiko-to (Xiao-Chai-Hu-Tang) and crude saikosaponins inhibit hepatitis B virus in a stable HBV-producing cell line Am J Chin Med 2007 35 341 351 10.1142/S0192415X07004862 17436373

[B14] Lee JK Kim JH Shin HK Therapeutic effects of the oriental herbal medicine Sho-saiko-to on liver cirrhosis and carcinoma Hepatol Res 2011 41 825 837 10.1111/j.1872-034X.2011.00829.x 21682829

[B15] Timmermans S Mauck A The promises and pitfalls of evidence-based medicine Health Aff (Millwood) 2005 24 18 28 10.1377/hlthaff.24.1.18 15647212

[B16] Eddy DM Evidence-based medicine: a unified approach Health Aff (Millwood) 2005 24 9 17 10.1377/hlthaff.24.1.9 15647211

[B17] Tang JL Liu BY Ma KW Traditional Chinese medicine Lancet 2008 372 1938 1940 10.1016/S0140-6736(08)61354-9 18930523

[B18] Kang BH Kim YB Lee HS Kim YH Im WJ Ha CS Background data on hematology, blood, biochemistry and organ weights for 2 weeks and 4 weeks repeated dose toxicity studies using Sprague–Dawley (SD) rat Lab Anim Res 2004 20 134 140

[B19] Michael JD The toxicologist pocket handbook 2008 USA: Informa Healthcare, CRC press

[B20] Nishimori F Yamazaki K Jin Y Yoshimura N Tsukimoto K Beppu H Ichioka M Yoshizawa Y Pneumonitis induced by the drug ougon Nihon Kokyuki Gakkai Zasshi 1999 37 396 400 10410542

[B21] Ishizaki T Sasaki F Ameshima S Shiozaki K Takahashi H Abe Y Ito S Kuriyama M Nakai T Kitagawa M Pneumonitis during interferon and/or herbal drug therapy in patients with chronic active hepatitis Eur Respir J 1996 9 2691 2696 10.1183/09031936.96.09122691 8980988

[B22] Nakagawa A Yamaguchi T Takao T Amano H Five cases of drug-induced pneumonitis due to Sho-saiko-to or interferone-alpha or both Nihon Kyobu Shikkan Gakkai Zasshi 1995 33 1361 1366 8821988

[B23] Chen WC Kuo TH Tzeng YS Tsai YC Baicalin induces apoptosis in SW620 human colorectal carcinoma cells in vitro and suppresses tumor growth in vivo Molecules 2012 29 3844 3857 22456615 10.3390/molecules17043844 PMC6268256

[B24] Guan Y Li FF Hong L Yan XF Tan GL He JS Dong XW Bao MJ Xie QM Protective effects of liquiritin apioside on cigarette smoke-induced lung epithelial cell injury Fundam Clin Pharmacol 2012 26 473 483 10.1111/j.1472-8206.2011.00956.x 21631586

[B25] Li XL Zhou AG Zhang L Chen WJ Antioxidant status and immune activity of glycyrrhizin in allergic rhinitis mice Int J Mol Sci 2011 12 905 916 10.3390/ijms12020905 21541033 PMC3083680

[B26] Xie Y Lu W Cao S Jiang X Yin M Tang W Preparation of bupleurum nasal spray and evaluation on its safety and efficacy Chem Pharm Bull 2006 54 48 53 10.1248/cpb.54.48 16394548

[B27] Yimam M Zhao Y Ma W Jia Q Do SG Shim JH 90-day oral toxicity study of UP446, a combination of defined extracts of Scutellaria baicalensis and Acacia catechu, in rats Food Chem Toxicol 2010 48 1202 1209 10.1016/j.fct.2010.02.011 20171255

[B28] National Toxicology Program Toxicology and carcinogenesis studies of ginseng (CAS No. 50647-08-0) in F344/N rats and B6C3F1 mice (gavage studies) Natl Toxicol Program Tech Rep Ser 2011 567 1 149 21921964

[B29] van Miert, ASJPAM The use in animals of drugs licensed for human use only Comparative Veterinary Pharmacology, Toxicology and Therapy 1986 Boston: MTP press 489 500

[B30] Manimaran A Sarkar SN Sankar P Toxicodynamics of subacute co-exposure to groundwater contaminant arsenic and analgesic-antipyretic drug acetaminophen in rats Ecotoxicol Environ Safety 2010 73 94 100 10.1016/j.ecoenv.2009.09.005 19782400

